# Comparative Efficacy of Ormeloxifene Versus Norethisterone Acetate in the Management of Abnormal Uterine Bleeding: A Randomized Controlled Trial

**DOI:** 10.7759/cureus.96844

**Published:** 2025-11-14

**Authors:** Vasundhara Gawande, Varsha Kose, Anuja Bhalerao

**Affiliations:** 1 Department of Obstetrics and Gynaecology, NKP Salve Institute of Medical Sciences and Research Centre, Nagpur, IND; 2 Department of Obstetrics and Gynaecology, NKP Salve Institute of Medical Sciences and Research Centre, Lata Mangeshkar Hospital, Nagpur, IND

**Keywords:** abnormal uterine bleeding, endometrial thickness, hemoglobin, norethisterone, ormeloxifene, pbac, randomized controlled trial

## Abstract

Background

Abnormal uterine bleeding (AUB) impacts women’s health and quality of life globally. Pharmacological management has prioritized hormonal agents such as norethisterone acetate; however, selective estrogen receptor modulators such as ormeloxifene have emerged as potential alternatives. Hence, this study aimed to assess and compare the effectiveness and safety of ormeloxifene and norethisterone acetate in women with AUB.

Methodology

This parallel, open-label, randomized controlled trial conducted at a tertiary center in central India included 60 women aged 21-47 years with AUB. Thirty participants each were assigned to ormeloxifene (60 mg twice weekly for 12 weeks, followed by once weekly for 12 weeks) or norethisterone acetate (5 mg twice daily for 21 days per cycle for six cycles). Primary endpoints included changes in pictorial blood loss assessment chart (PBAC) scores, hemoglobin concentration, and endometrial thickness (ET). Safety and the need for a hysterectomy were also assessed.

Results

Both groups were demographically comparable. The mean reduction in PBAC score at six months favored the ormeloxifene arm (-124.63 ± 14.65 vs. -103.73 ± 18.33, p < 0.001). The mean hemoglobin increase was significantly greater with ormeloxifene (2.26 ± 0.43 g/dL vs. 1.91 ± 0.66 g/dL, p = 0.019). ET reduction at six months was also superior in the ormeloxifene group (-8.90 ± 3.74 mm versus -6.20 ± 2.66 mm, p = 0.002). Side effects were infrequent and well-controlled, and surgical intervention was rarely required.

Conclusions

Ormeloxifene demonstrates superior efficacy in reducing menstrual blood loss, improving anemia, and suppressing endometrial proliferation in women with AUB compared to norethisterone acetate, with an acceptable safety profile.

## Introduction

Abnormal uterine bleeding (AUB) is a widespread gynecological problem that disrupts women’s health and quality of life. AUB refers to menstrual cycle irregularities in frequency, timing, duration, or volume that occur outside of pregnancy. Approximately one in three women may encounter AUB during their lives, with peaks at puberty and around menopause. Normally, menstrual cycles last from 24 to 38 days, with bleeding for two to seven days, and total blood loss between 5 and 80 mL; any deviation is considered AUB [1].

AUB can arise from many causes, spanning all age groups. These include structural conditions, such as fibroids, polyps, and endometrial hyperplasia, and non-structural issues, such as ovulatory dysfunction, coagulopathies, and endometrial disorders. The PALM-COEIN system organizes AUB causes into structural (polyp, adenomyosis, leiomyoma, malignancy/hyperplasia) and non-structural (coagulopathy, ovulatory dysfunction, endometrial, iatrogenic, and unclassified) categories [2]. In India, AUB affects 17% to 33% of reproductive-age women, creating a significant healthcare burden and emphasizing the need for effective, accessible treatments [3,4].

Initial management of AUB typically involves drug therapy, using either hormonal agents, such as progestogens and combined oral contraceptives, or non-hormonal treatments, such as antifibrinolytics [5]. Norethisterone acetate, a synthetic progestin, is effective in reducing menstrual bleeding and regularizing cycles and remains a gold standard for ovulatory AUB [6,7]. Despite its benefits, it can cause adverse effects, including breakthrough bleeding, cardiovascular risks, and fluid retention, particularly with prolonged use [8].

Recently, ormeloxifene, a selective estrogen receptor modulator, has been gaining attention as a non-hormonal alternative [9-11]. It exhibits estrogenic effects in some tissues and anti-estrogenic effects in others, acting as an antagonist in the uterus and breast while being estrogenic on the vagina and systems such as the cardiovascular and central nervous systems [8]. Ormeloxifene’s capacity to reduce endometrial proliferation and menstrual blood loss, combined with a favorable safety profile, makes it a potential alternative to traditional progestins [12].

Few head-to-head studies, especially in central India, have directly compared norethisterone acetate and ormeloxifene in AUB management, with mixed results regarding superior efficacy [9-11,13]. Given the push toward less invasive, organ-preserving treatments, it is essential to evaluate which of these medications offers better outcomes and fewer side effects. This context sets the stage for a randomized controlled trial comparing both drugs in Indian women with AUB, aiming to inform optimal therapeutic choices and improve patient care.

The objective of the study was to assess and compare the effectiveness and safety of ormeloxifene and norethisterone acetate in women with AUB with respect to menstrual blood loss, hemoglobin concentration, and endometrial thickness (ET). Secondary outcomes included the incidence and profile of drug-related adverse events, need for additional medical management, and rates of surgical intervention (hysterectomy).

## Materials and methods

This study was designed as a randomized controlled trial conducted at the Department of Obstetrics and Gynaecology in a tertiary care center following approval from the Institutional Ethics Committee (approval number: 46/2023). This study was prospectively registered with the Clinical Trials Registry - India (CTRI) (registration number: CTRI/2024/12/078353), in accordance with national guidelines. Institutional Ethics Committee approval was obtained before registering the trial, ensuring compliance with ethical standards. The study population included women between 21 and 47 years of age who presented with AUB as per the PALM-COEIN classification and who sought treatment from July 2023 to May 2025.

Women who were willing to participate, maintain menstrual records, and attend regular follow-up visits were included in the study. Exclusion criteria were postmenopausal bleeding, known genital malignancy, fibroids larger than 3 cm, polycystic ovarian syndrome, uterine size greater than 12 weeks, hepatic or renal dysfunction, heart disease, allergy to the study medications, and puberty menorrhagia.

Sample size and randomization

The sample size was calculated using the formula for comparing two independent means, considering a significance level (α) of 0.05, a power of 80%, a pooled standard deviation (SD), and the expected difference in means. According to a previously published article, in the ormeloxifene group, the mean pictorial blood loss assessment chart (PBAC) score decrease was 33.66 with an SD of 13.43, and in the norethisterone acetate group, the PBAC score decrease was 57.81 with an SD of 14.3 [14]. This resulted in 30 participants per group to reliably detect a difference in PBAC score reduction between treatments.

Eligible participants were recruited consecutively and randomized into two groups (A: ormeloxifene, B: norethisterone) by block randomization with computer-generated allocation using the RALLOC software [15]. This trial was open-label, and neither the subjects nor the investigators were blinded to treatment assignments.

Interventions

All enrolled women underwent a comprehensive baseline assessment, including medical history, general and gynecological examination, and relevant investigations. Endometrial sampling for histopathology was performed if suggested by clinical findings. Participants were educated on the use of the PBAC to objectively monitor menstrual blood loss by recording the number and saturation of sanitary products and noting any clots [16,17].

In the study, treatment adherence was monitored by daily and weekly phone reminders to encourage medication intake. Participants also maintained medication diaries and provided self-reports during follow-up calls. These straightforward methods helped ensure compliance and accurate tracking of drug use throughout the trial.

Group A participants received ormeloxifene 60 mg twice weekly for 12 weeks, followed by once weekly for another 12 weeks. Group B received norethisterone acetate 5 mg twice daily for 21 days each cycle for six cycles. Both groups received iron supplementation (60 mg twice daily) throughout the study period. If active bleeding persisted despite treatment, adjunctive tranexamic acid was administered as required.

**Figure 1:**
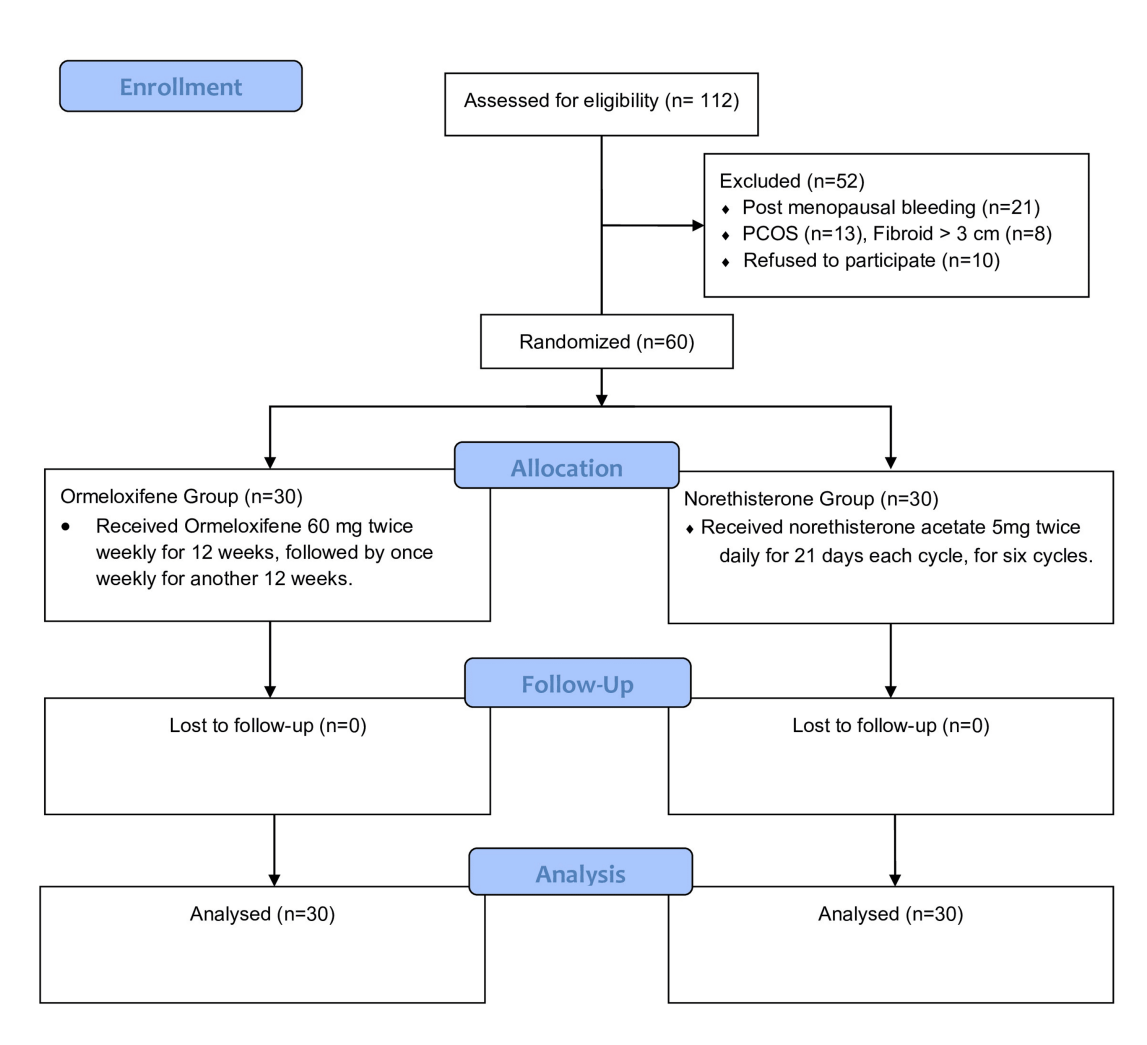
CONSORT diagram showing the flow of participants through each stage of the randomized trial.

The CONSORT flowchart (Figure 1) summarizes participant enrollment, allocation, treatment, follow-up, and analysis, ensuring transparency of the trial process and participant disposition.

Evaluation and outcome measures

The primary efficacy outcomes were reduction in menstrual blood loss, rise in hemoglobin concentration, and decrease in ET on transvaginal ultrasound. These parameters were recorded at baseline, one month, three months, and six months. Secondary outcomes included the incidence and profile of drug-related adverse events, need for additional medical management, and rates of surgical intervention (hysterectomy).

Adverse effects related to both drugs, including amenorrhea, oligomenorrhea, hypomenorrhea, headaches, nausea, and withdrawal bleeding, were documented at each visit. Participants not responding to medical therapy or experiencing intolerable side effects could be discontinued from their assigned group and considered for alternative management [18,19].

Data management and analysis

Participant data were securely recorded in case files and subsequently transcribed into a Microsoft Excel (Microsoft Corp., Redmond, WA, USA) database. Data quality was ensured through routine validation and verification by study supervisors. For statistical analysis, the data were coded and analyzed using STATA version 10.1 (StataCorp., College Station, TX, USA). Descriptive statistics were used to summarize the participants’ baseline characteristics, while independent t-tests and chi-square tests assessed differences between groups. Differences were deemed statistically significant if the p-value was below 0.05.

## Results

Most participants were between 31 and 40 years old, with 37 (61.7%) cases overall: 21 (70%) cases in group A, and 16 (53.4%) cases in group B. The second most represented age group was 41-47 years. The mean age was 39.07 ± 4.14 years in group A and 40.37 ± 3.66 years in group B. Body mass index (BMI) distribution was also comparable. Most women were classified as Obese I (BMI of 25-29.9 kg/m²), with 40 (66.7%) cases overall in this category. Socioeconomic status distribution showed a predominance of the upper-lower class (41 of 60 cases, 68.3%) in both groups. Multiparous women dominated the study population, with G2-G3 comprising 41 of 60 (68.3%) cases (Table 1).

**Table 1:** Baseline characteristics of the study participants. BMI: body mass index; SES: socioeconomic status; G: gravida

Variables		Ormeloxifene	Norethisterone	Total (N = 60)	P-value
		(n = 30)	(n = 30)		
Age group (years)	21–30	2 (6.7%)	1 (3.3%)	3 (5%)	Chi-square value = 2.81, p = 0.245
	31–40	21 (70.0%)	16 (53.4%)	37 (61.7%)	
	41–47	7 (23.3%)	13 (43.3%)	20 (33.3%)	
BMI category (kg/m²)	Normal (18.5–22.9)	2 (6.7%)	3 (10.0%)	5 (8.3%)	Chi-square value = 0.22, p = 0.92
	Overweight (23–24.9)	7 (23.3%)	7 (23.3%)	14 (23.3%)	
	Obese I (25–29.9)	21 (70.0%)	19 (63.3%)	40 (66.7%)	
	Obese II (>30.0)	0 (0.0%)	1 (3.3%)	1 (1.7%)	
SES category	Lower middle	5 (16.7%)	3 (10.0%)	8 (13.3%)	Chi-square value = 1.34, p = 0.532
	Upper lower	21 (70.0%)	20 (66.7%)	41 (68.3%)	
	Upper middle	4 (13.3%)	7 (23.3%)	11 (18.3%)	
Gravida category	Primigravida	5 (16.7%)	4 (13.3%)	9 (15.0%)	Chi-square value = 0.535, p = 0.855
	G2–G3	21 (70.0%)	20 (66.7%)	41 (68.3%)	
	G4–G5	4 (13.3%)	6 (20.0%)	10 (16.7%)	

Clinical presentation and baseline features

Heavy menstrual bleeding was the leading complaint (46.7% overall), followed by prolonged bleeding (40.0%) and infrequent bleeding (30.0%). Other symptoms included frequent, postcoital, and intermenstrual bleeding, distributed similarly between groups (Table 2).

**Table 2:** Presenting complaints of the study participants and PALM-COEIN classification of cause. For causes of AUB in the two groups, the chi-square statistic is 4.1825, with a p-value of 0.38. AUB: abnormal uterine bleeding; PALM-COEIN: polyp, adenomyosis, leiomyoma, malignancy and hyperplasia, coagulopathy, ovulatory dysfunction, endometrial causes, iatrogenic causes, and not yet classified

Variables		Ormeloxifene	Norethisterone	Total (N = 60)
		(n = 30)	(n = 30)	
Presenting complaints	Heavy menstrual bleeding	15 (50%)	13 (43.33%)	28 (46.7%)
	Prolonged bleeding	10 (33.33%)	14 (46.67%)	24 (40.0%)
	Infrequent bleeding	9 (30%)	9 (30.00%)	18 (30.0%)
	Frequent bleeding	4 (13.33%)	2 (6.67%)	6 (10.0%)
	Postcoital bleeding	3 (10%)	6 (20.00%)	9 (15.0%)
	Intermenstrual bleeding	2 (6.67%)	5 (16.67%)	7 (11.7%)
Cause of AUB (PALM-COEIN) [2]	Polyp	1 (3.3)	5 (16.7)	6 (10.0%)
	Adenomyosis	9 (30.0)	9 (30.0)	18 (30.0%)
	Leiomyoma	12 (40.0)	7 (23.3)	19 (31.7%)
	Malignancy and hyperplasia	2 (6.7)	3 (10)	5 (8.33%)
	Ovulatory	6 (20.0)	6 (20.0)	12 (20.0%)

As per the PALM-COEIN classification, leiomyoma (L) and adenomyosis (A) were the most frequent etiologic subtypes, totaling 19 (31.7%) and 18 (30%), respectively. The groups were statistically similar in the distribution of these AUB causes (chi-square statistic = 4.1825, p = 0.38) (Table 2). No significant differences were observed for any baseline measure, confirming successful randomization (Table 3).

**Table 3:** Baseline demographics and clinical characteristics by the study group. BMI: body mass index; PBAC score: pictorial blood assessment chart score

Characteristic	Ormeloxifene	Norethisterone	t-value	P-value
	(n = 30)	(n = 30)		
Age (years)	39.07 ± 4.14	40.37 ± 3.66	-1.930	0.203
BMI (kg/m²)	26.13 ± 2.03	25.81 ± 2.32	0.578	0.565
Menstrual cycle frequency (days)	35.30 ± 4.84	35.43 ± 6.21	-0.093	0.926
Menstrual bleeding duration (days)	7.87 ± 1.33	8.13 ± 1.41	-0.754	0.454
PBAC score	177.63 ± 16.33	178.33 ± 8.92	-0.206	0.837
Endometrial thickness (mm)	12.11 ± 5.66	12.12 ± 5.54	-0.005	0.996
Hemoglobin (g/dL)	9.04 ± 0.70	9.31 ± 0.83	-1.345	0.184

Hemoglobin concentration over time

**Figure 2:**
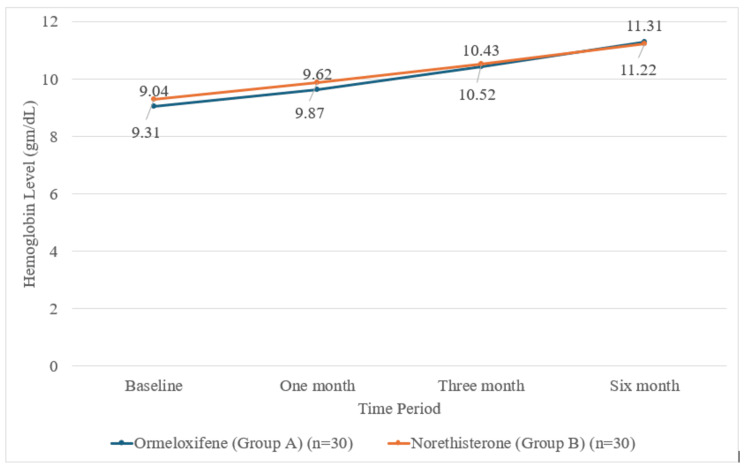
Hemoglobin concentration over time by the study group.

Both interventions resulted in progressive improvement in hemoglobin levels over six months. At one month, mean hemoglobin increased to 9.62 ± 0.77 g/dL in group A and 9.87 ± 0.82 g/dL in group B. By three months, hemoglobin levels reached 10.43 ± 0.74 g/dL in group A and 10.52 ± 0.76 g/dL in group B. At six months, group A had a hemoglobin value of 11.31 ± 0.71 g/dL, and group B had a hemoglobin level of 11.22 ± 0.89 g/dL (Figure 2).

While both groups made gains, the increase in hemoglobin from baseline was significantly greater in the ormeloxifene arm at three months (t-value = 2.286, p = 0.026) and at six months (t-value = 2.421, p = 0.019). This highlights ormeloxifene’s greater impact on anemia correction over prolonged therapy.

Menstrual blood loss (PBAC score)

**Figure 3:**
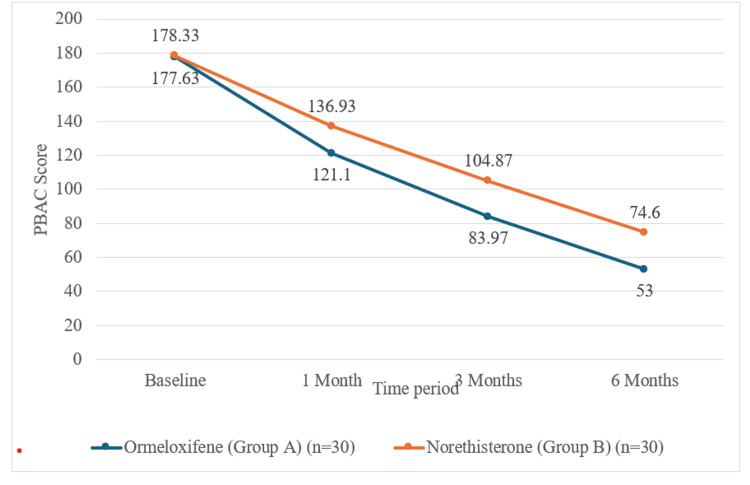
PBAC score over time by the study group. PBAC: pictorial blood loss assessment chart

Initial PBAC scores were nearly identical (group A: 177.63 ± 16.33, group B: 178.33 ± 8.92). Substantial reductions in menstrual blood loss were seen in both groups, but decreases were consistently greater and highly significant for ormeloxifene at each follow-up (Figure 3).

At one month, PBAC dropped to 121.10 ± 21.91 in group A and 136.93 ± 13.29 in group B; the difference was significant with a t-value of -3.384 and a p-value of 0.001. The mean PBAC score showed a significant decline in group A (56.53 ± 15.37) compared to group B (41.40 ± 13.29), with statistical analysis yielding a t-value of -4.079 and a p-value <0.001.

At three months, the mean PBAC score was significantly lower in group A (83.97 ± 19.30) compared to group B (104.87 ± 14.61), with statistical testing revealing a t‑value of -4.729 (p < 0.001). Analysis of PBAC score reduction from baseline further highlighted a greater decline in group A (-93.67 ± 17.63) than in Group B (-73.47 ± 14.16), and this difference was statistically significant (t = -4.892, p < 0.001).

At six months, group A showed a significantly lower mean PBAC score (53.00 ± 20.35) compared with group B (74.60 ± 16.53; t = -4.513, p < 0.001). The reduction from baseline was also greater in group A (-124.63 ± 14.65 vs. -103.73 ± 18.33; t = -4.878, p < 0.001), indicating superior treatment efficacy. These data confirm that ormeloxifene offers superior control of menstrual blood loss versus norethisterone in women with AUB.

Endometrial thickness

**Figure 4:**
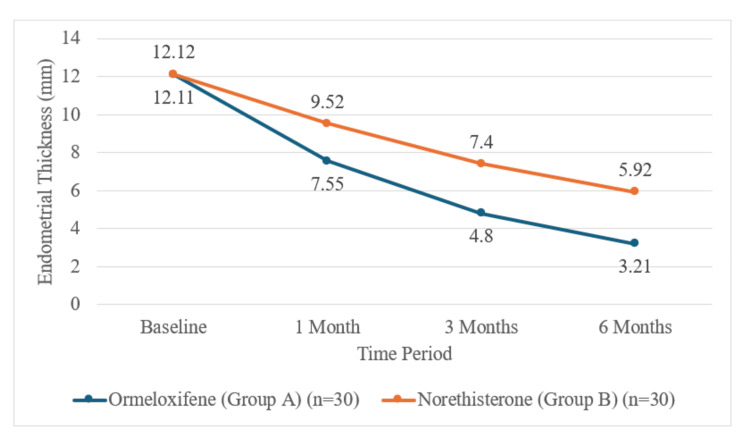
Endometrial thickness over time by the study group.

Both treatments significantly reduced ET, but the effect was more notable and achieved faster with Ormeloxifene (Figure 4).

At one month, the mean reduction from baseline was greater in group A (-4.56 ± 3.20 mm) compared with group B (-2.60 ± 1.84 mm). The difference in mean reduction was statistically significant (t = -2.910, p = 0.005). The magnitude of this difference widened further at both three and six months.

At the three-month follow‑up, the mean ET reduction was greater in group A (-7.31 ± 3.56 mm) compared with group B (-4.72 ± 2.33 mm), and the difference was statistically significant (t = -3.342, p = 0.001).

At the six-month follow-up, the mean ET was significantly lower in group A (3.21 ± 3.07 mm) compared with group B (5.92 ± 5.70 mm; t = -2.291, p = 0.027). The mean reduction from baseline was also greater in group A (-8.90 ± 3.74) than in group B (-6.20 ± 2.66), a statistically significant difference (t = -3.229, p = 0.002).

Adverse effects

At six months, a total of seven (23.3%) women in the Ormeloxifene group and two (6.7%) women in the norethisterone group reported side effects, though this difference did not reach statistical significance (chi-square with Yates correction value = 2.09, p = 0.148). The most frequent adverse effects in group A were amenorrhea (four cases), headache (six cases), and nausea (five cases). Most women in each group completed therapy without significant side effects, and no dropouts were reported due to adverse events.

Surgical outcomes

Hysterectomy was required in just three cases: one in group A (ormeloxifene) and two in group B (norethisterone), representing a low surgical conversion rate (5.0% of the total), with no significant difference between groups (p = 1.000).

## Discussion

AUB remains a major cause of gynecological morbidity, affecting women’s health and quality of life across the globe, with the highest incidence seen in the reproductive and perimenopausal years. In this randomized controlled trial, the efficacy and safety of ormeloxifene were directly compared to those of norethisterone acetate.

Participant profile

The current study included 60 women aged 21-47 years, with the majority (over 60%) in the 31-40-year age group. This age group is well-represented in similar Indian studies, including those by Fatima and Siddiqua (2024) [20], Meena et al. (2022) [13], and Talukdar et al. (2016) [12], which note that AUB often peaks in women’s mid-reproductive years. Multigravida and multiparity were common, echoing findings from Karmakar et al. (2016) [7], Verma (2016) [21], and Nayak et al. (2017) [22], who reported higher AUB incidence in women who have completed childbearing, likely contributing to better compliance with oral medical management. Most participants were in the overweight or Obese I BMI category, reflecting shifting lifestyle factors in urban India and paralleling trends reported by Singh et al. (2019) [23] and Misra and Srivastava (2021) [24].

Clinical presentation

Heavy menstrual bleeding and prolonged cycles were the most common complaints at presentation (nearly 47% and 40%, respectively), consistent with Indian and international data, which point to menorrhagia and irregular bleeding patterns as the most frequent AUB symptoms [25]. The application of the PALM-COEIN system showed leiomyoma (L) and adenomyosis (A) as leading causes of AUB, followed by ovulatory dysfunction (O). This mirrors reports from Misra and Srivastava (2021) [24] and Sinha et al. (2018) [26], highlighting a mix of structural and functional etiologies in typical Indian clinical practice.

Improvements in hemoglobin

Correction of anemia is a fundamental treatment goal for AUB. Both ormeloxifene and norethisterone significantly improved hemoglobin levels over six months, but ormeloxifene demonstrated a statistically greater mean rise at three months (1.39 g/dL vs. 1.26 g/dL, p = 0.026) and six months (2.26 g/dL vs. 1.91 g/dL, p = 0.019). This is concordant with Fatima and Siddiqua (2024) [20], Agrawal et al. (2019) [11], and Surabhi and Chandra Mouli (2018) [8], all of whom found a greater or more rapid improvement in hemoglobin with ormeloxifene compared to progestins, even if some studies found the absolute difference to not always be statistically significant. The importance of this finding is highlighted by Gett and Singh (2018) [27], who noted a 31.5% hemoglobin rise with ormeloxifene therapy, substantially reducing the burden of anemia in these women.

Menstrual blood loss reduction (PBAC scores)

Reduction in menstrual blood loss, assessed objectively by the PBAC score, was significantly better with ormeloxifene. The mean scores fell from 177.6 to 53.0, compared to a decrease from 178.3 to 74.6 in the norethisterone group (p < 0.001). These findings are consistent across multiple Indian and international comparative trials. Talukdar et al. (2016) [12], Patil et al. (2023) [14], and Choudhary and Gupta (2018) [28] all reported that ormeloxifene achieves a faster, larger, and more sustained drop in blood loss than norethisterone. Fatima and Siddiqua (2024) [20] observed an 80.2-point PBAC drop with ormeloxifene versus 53.7 with norethisterone at six months, while Gett and Singh (2018) [27] found reductions of 66.53% and 31.38%, respectively, confirming that the antiproliferative action of selective estrogen receptor modulators more effectively halts excessive menstrual flow.

Reduction in endometrial thickness

ET is a surrogate for treatment efficacy, especially in non-structural AUB. In this trial, ET reduced significantly more with ormeloxifene at every post-baseline point, culminating in a mean decrease of 8.9 mm compared to 6.2 mm with norethisterone at six months (p = 0.002). Fatima and Siddiqua (2024) [20], Meena et al. (2022) [13], and Choudhary and Gupta (2018) [28] similarly showed that ormeloxifene leads to a greater and faster reduction in ET, believed to be due to its antiestrogenic effect on endometrial tissue. Gett and Singh (2018) [27] also identified a greater percentage reduction in ET with ormeloxifene (25.2% vs. 11.9%), making it particularly useful for preventing recurrence of abnormal bleeding.

Side effects and safety profile

Ormeloxifene was associated with a slightly higher incidence of minor side effects at six months (23.3% vs. 6.7%), but this difference did not reach statistical significance and included mostly amenorrhea, headache, and nausea. Similar trends were reported by Karmakar et al. (2016) [7] and Surabhi and Chandra Mouli (2018) [8], who found that side effects such as amenorrhea were rarely a cause for discontinuation and often welcomed by multiparous women for whom fertility is not a priority. Shahab et al. (2014) [29] reported a higher rate of amenorrhea and irregular periods in ormeloxifene users but found that overall compliance remained strong. Notably, no participants in either group withdrew from the study due to adverse effects.

Surgical conversion (need for hysterectomy)

Although some women with AUB ultimately require surgery, especially for persistent or structural disease, the need for hysterectomy in both study arms was low (3.3% for ormeloxifene and 6.7% for norethisterone, p = 1.000). This supports prior findings that effective drug therapy can avert surgical intervention in most cases; for instance, Kriplani et al. (2009) [30] and Shahab et al. (2014) [29] found higher surgical rates (10-21%), likely reflecting differences in sample or treatment duration. Most surgical cases in this study were due to hyperplasia, leiomyoma, or adenomyosis.

Study strengths and limitations

This work benefits from a robust, randomized design and well-matched groups but is limited by single-center recruitment and modest sample size. Larger multicenter trials would offer increased power and external validity. However, the results are bolstered by extensive consistency with diverse published data and support the utility of ormeloxifene as a first-line therapy for AUB.

## Conclusions

Both ormeloxifene and norethisterone acetate proved effective in reducing menstrual blood loss and correcting anemia among women with AUB, but ormeloxifene consistently demonstrated greater improvements across all primary endpoints. Over the six-month trial, women receiving ormeloxifene experienced a larger and more rapid decline in PBAC scores, a greater rise in hemoglobin concentration, and a more substantial reduction in ET. The medication was well-tolerated overall, with reported side effects being mild and not leading to discontinuation, and major complications were rare. These outcomes underline ormeloxifene’s robust efficacy and safety profile, confirming its suitability as a first-line option for non-structural AUB. The low rate of surgical intervention observed in both groups highlights the potential of effective medical therapies to reduce the need for hysterectomy in Indian women suffering from AUB. These findings reinforce current guidelines that advocate for individualized and conservative management before considering surgical options for AUB and support the wider adoption of ormeloxifene as a preferred therapy in suitable cases. The study findings should be interpreted in the light of the smaller sample size and the availability of six-month follow-up data only.
